# Prone positioning in acute respiratory distress syndrome after abdominal surgery

**DOI:** 10.1186/cc14323

**Published:** 2015-03-16

**Authors:** S Gaudry, S Tuffet, AC Anne-Claire, N Zucman, S Msika, M Pocard, D Payen, D Dreyfuss, JD Ricard

**Affiliations:** 1 Hôpital Louis Mourier, Colombes, France; 2 Hôpital Lariboisière, Paris, France

## Introduction

Prone positioning has been used for many years as an alveolar recruitment strategy in acute respiratory distress syndrome (ARDS). Prone positioning in ARDS improves oxygenation and demonstrated recently its effectiveness on prognosis. Extrapulmonary etiologies of ARDS include abdominal emergencies. In cases of severe hypoxemia in the early postoperative period, intensivists discuss prone positioning based on the risk/benefit ratio.

## Methods

We conducted a retrospective two-center study of 5 years. The aim was to compare the prevalence of surgical complication potentially related to prone positioning between patients who had at least one prone positioning session and patients that remained in a supine position after abdominal surgery. Patients with ARDS in a context of recent (<7 days) abdominal surgery (except laparoscopy) were included. The primary outcome was the number of patients who had at least one surgical complication potentially related to prone positioning. We defined *a priori *these complications: scar dehiscence, abdominal compartment syndrome, stoma leakage, stoma necrosis, scar necrosis, wound infection, displacing of a drainage system, removal of gastro- or jejunostomy feeding, digestive fistula, evisceration.

## Results

We identified 43 patients with postoperative ARDS (62 ± 8 years, SAPS II 50 ± 13), among whom 34 (79%) had emergent surgery. Fifteen patients had at least one stoma after surgery. Nineteen patients (44%) had at least one prone positioning session (number of sessions: 2 (1 to 3)). At baseline, prone group patients had minimum PaO_2_/FiO_2 _ratio lower than the supine group (77 ± 23 vs. 110 ± 46 mmHg, *P *= 0.005). Plateau pressure was higher in the prone group (28 ± 4 vs. 23 ± 5 cmH_2_O, *P *= 0.002). The first prone positioning session significantly increased the PaO_2_/FiO_2_ ratio: 106 ± 52 vs. 192 ± 90 mmHg (*P *= 0.001). Mean duration of the first prone positioning session was 20 ± 10 hours. In the prone group, 11 patients (58%) had at least one surgical complication, in comparison with nine (38%) in the supine group (*P *= 0.2). These complications resulted in revision surgery for two (10%) patients in the prone group and two (8%) in the supine group (*P *= 0.8). Mortality in the ICU was respectively 42% and 38% in prone group and supine group (*P *= 0.8).

## Conclusion

These preliminary results confirm the effectiveness of prone positioning in terms of oxygenation in ARDS after abdominal surgery without significant increase in surgical complications and no effect on the need for surgical revisions. Hence, if necessary, clinicians should not refrain from proning patients with postabdominal surgery ARDS.

